# 10 years’ cultural variation of Croatia from pre to post accession: the changed value and unchanged cultural position

**DOI:** 10.3389/fsoc.2025.1487913

**Published:** 2025-07-21

**Authors:** Chunyan Wang, Ivica Bakota, Ivana Buljan, Yuhong Shang

**Affiliations:** ^1^School of Languages, Shanghai University of International Business and Economics, Shanghai, China; ^2^Institute for Global and Area Studies, Capital Normal University, Beijing, China; ^3^Faculty of Humanities and Social Sciences, University of Zagreb, Zagreb, Croatia; ^4^School of International Business, Shanghai University of International Business and Economics, Shanghai, China

**Keywords:** cultural value, Hofstede, Croatia, transition, cultural distance, the EU integration

## Abstract

**Introduction:**

Croatia’s accession to the European Union (EU) in 2013 completed its institutional integration; however, the alignment of its social and cultural values with those of other EU member states remains an ongoing process.

**Methods:**

Utilizing Hofstede’s cultural dimensions framework and data from rounds 5 and 10 of the European Social Survey (ESS), this study examines the evolution of Croatia’s value orientations from 2010 to 2020 and assesses whether the cultural value gap between Croatia and other EU members has narrowed.

**Results:**

The study identifies significant cultural shifts: the decline in Masculinity, Uncertainty Avoidance, and Power Distance indexes, alongside an increase in Individualism. These shifts are in the same direction with those of the old EU members. However, despite these changes, Croatia’s cultural value distance from other EU members has largely remained constant. Particularly, Power Distance index in Croatia is persistently higher than the average level.

**Discussion:**

These findings suggest that the EU should strengthen its common values within the newly accessed members. Policies aimed at encouraging participation in EU-wide cultural and economic projects may also bridge cultural divides. The study contributes to an understanding of cultural change in post-transition societies and their implications for EU integration.

## 1 Introduction

Cultural values guide the selection or evaluation of actions, policies, and events. Using their values, people in a culture judge what is good or bad and what actions should or should not be taken (Schwartz, 2004; Hofstede, 2001). These values change alongside fundamental shifts in socioeconomic and political structures (Pehe and Sommer, 2022). In the European context, a country’s accession to the European Union (EU) represents a significant turning point in the value formation process (Akaliyski et al., 2021). The impact of social institutions prior to the transition makes it unlikely that a lasting value equilibrium will be quickly established. Therefore, it is crucial to periodically assess the value orientations of transitional countries (Kolman et al., 2003). Such studies hold not only practical significance but also considerable theoretical importance, as these countries, still in the process of rebuilding their societies, serve as vast social laboratories for observing the interaction between cultural heritage and new societal institutions.

Croatia transited from a part of pre-Yugoslavia to an EU member and was acceded to the EU in 2013, signifying the completion of institutional transition. However, there has been limited research on the variation in Croatia’s values in the transition process, although scholars criticized the transition for lagging in the socio-cultural aspect (Fink-Hafner, 2008). Political backlash has been observed after the removal of pre-membership conditionality (Čepo, 2020). For instance, just six months after joining the EU, Croatians rejected homosexual marriage and constitutionally defined marriage as a heterosexual union, a clear violation of the common values endorsed by the EU (Juroš et al., 2020).

Our objective is to empirically study whether the cultural values of Croatian citizens have changed since Croatia became a new EU member and whether the cultural distance between Croatia and other EU countries has been reduced. Examining the shift in cultural values in Croatia is valuable for understanding whether its EU membership has contributed to the cultivation of shared values among EU members. This study complements prior research on the impact of major sociopolitical events on the value-formation process (Egri and Ralston, 2004). From a practical standpoint, the study will assist EU policymakers in designing effective policies for strengthening common values and aid cross-cultural researchers and practitioners in better understanding the value orientations of new EU members.

The structure of the article is as follows: the literature section provides an overview of culture theory and the key factors influencing Croatian cultural transformation, including the EU’s cultural integration, the Croatian transition process, and socioeconomic development. Building on these factors, hypotheses regarding cultural variations are illustrated. The method part explains how we investigated changes in Croatian cultural values and their proximity to those of the old EU members during 2010–2020. In the subsequent sections, the primary findings and implications of the study are discussed. In the study, we argued that although Croatia experienced cultural value changes aligning with those of the old EU members, the cultural distance between Croatia and other EU members has not shortened as is expected after such a transition.

## 2 Literature review

### 2.1 Cultural value variation

Culture represents the collective configuration of societal values characteristic of a country’s population (Schwartz, 2004). A value is “a broad tendency to prefer certain states of affairs over others.” (Hofstede, 2001, p. 5). Cultural value evolves slowly with economic growth, industrialization, and globalization (Schwartz, 2004). Economic growth is associated with a pronounced rise in individualism, egalitarian values, tolerance, and trusting norms (Inglehart and Baker, 2000). Technological advances facilitate this transition by altering daily practices and opportunities, thereby reshaping societal norms and individual expectations (Schwartz, 2004). Globalization in the past century has promoted cultural convergence toward dominant global values, such as individualism and tolerance (Beugelsdijk et al., 2015).

Cultural value changes with shifts in socioeconomic and political structures (Pehe and Sommer, 2022). In the European context, a significant turning point in value formation is a country’s accession to the EU (Akaliyski et al., 2021). Joining the EU meant adopting European values, potentially leading to value convergence toward EU-wide ideals like individualism and egalitarianism (Turkina and Surzhko-Harned, 2014). However, this process can also trigger resistance and a resurgence of traditional or conservative values reflecting national identity and skepticism (Akaliyski et al., 2021).

### 2.2 Cultural value integration in the EU

Promoting shared cultural values is a key priority for the EU, especially as each enlargement increases cultural diversity (Gerhards, 2010). The Consolidated Treaty on the European Union (2016) states that member states must uphold values such as pluralism, non-discrimination, tolerance, justice, solidarity, and gender equality. The EU promotes these values primarily by encouraging the free movement of people and goods, aligning societal structures, and reducing economic disparities among member states (Akaliyski, 2018).

Researchers have found that the EU has successfully promoted these values among member states. Oshri et al. (2015) showed that support for democratic values in new member states increased steadily with each year of EU membership. Akaliyski and Welzel (2020) found that between 1992 and 2008, the cultural gap between old and new member states narrowed significantly, with new members increasingly adopting the cultural norms of older ones.

However, some research differed in their findings as to whether the EU states are converging their cultural values. For example, Van Houwelingen et al. (2018) found cultural values were diverging among member states. The convergence or divergence of cultural distance within the EU states is conditional on the speed of the change of the old, new and prospective member states. If cultural values of these states change at different speeds, even if they are changing to the same direction, culture divergence would be the result.

### 2.3 Croatia transition and value changes

Croatia started its social and economic transition toward the EU since the late 1980’s. In the 1990s, its democratic transition coincided with state-building and war (Kasapović, 1996). The government and political elites in that period were preoccupied with the nation state building efforts. In 2001 Croatia began an official EU integration stage lasting for the next twelve years (2001–2013). During this period, Croatia steadily advanced toward the EU, carrying out all necessary reforms and fulfilling criteria for the accession.

The post-accession period since 2013 was characterized by the completion of the transition process as Croatia entered the Schengen zone and adopted the Euro as its national currency. During this time, Croatia developed a relatively open market economy, a multi-party democracy, and advanced rule of law standards. According to data from the World Bank (2022), Croatia’s GDP increased by approximately 3.4% annually over the decade, significantly higher than the EU’s average growth rate of 1.7%.

Research on the transition of cultural values in Croatia is limited and offers inconclusive findings. Rajh et al. (2016) found that Croatians are consistent with EU members in showing low power distance and high individualism. They attributed this change to globalization, which aligns people’s mindsets with those of advanced societies. Similarly, Akaliyski (2018) found that from 2001 to 2008, the newly accepted EU member states, including Croatia, significantly narrowed the cultural gap with older EU members. However, Dabić et al. (2023) examined value changes from 2002 to 2015 and noted that Croatia’s value formation had not adequately aligned with EU common principles and shared values.

### 2.4 The replication of Hofstede’s culture theory with the ESS

Various theories have been developed to study cultural values (e.g., Inglehart and Baker, 2000; House et al., 2004). Among them, Hofstede (1980, 2001) cultural framework is most influential, with over 50 million citations (Venkateswaran and Ojha, 2019). It has been widely applied in fields such as applied psychology (e.g., Zheng et al., 2025), human resource management (e.g., Muleya et al., 2023), and international trade (e.g., Harms and Shuvalova, 2020). Hofstede’s cultural dimensions have been proved to reflect real cultural differences (Kolman et al., 2003). As Hofstede’s theory is still widely used and referred to by researchers and practitioners across disciplines (Beugelsdijk and Welzel, 2018), we adopted it as the analytical framework in this study.

Considering the significant changes in cultural values worldwide, developing new cultural dimension indicators based on Hofstede’s theory is important (Taras et al., 2022; Hahn and Doh, 2025). Large-scale surveys such as the European Social Survey (ESS), have often been chosen to update Hofstede’s findings. The ESS contains basic socio-economic and demographic information of European countries. Combining Hofstede’s theory with ESS data has been profoundly explored (Kaasa et al., 2013, 2014; Kaasa, 2021; Kaasa and Welzel, 2023). Kaasa et al. (2013, 2014) twice combined Hofstede’s theory with ESS data in doing cross-culture study, and theoretically justified the combination of the two later (Kaasa, 2021).

To summarize, the literature review indicates that there has been little systematic analysis of the cultural value transition in Croatia. Kolman et al. (2003) emphasized the need for periodic assessments of value orientations in transitional countries. We aim to address this gap by examining Croatian cultural values from 2010 to 2020. This timeframe strategically captures the decade before and after accession, highlighting the fundamental reforms from a socialist society to an EU member state (Vukadinović, 2013). This study provides insights into the cultural transformation experienced and culture challenges faced by Croatia in the process of Europeanization.

### 2.5 The hypotheses of cultural value changes in Croatia

The following hypotheses are based on the influence of economic growth on cultural change in modern societies (Hofstede, 2001; Schwartz, 2004). More importantly we considered value shifts in contextual transformation process of Croatia (Pehe and Sommer, 2022).

#### 2.5.1 Power distance (PD) variation hypothesis

PD reflects the extent to which individuals in a society expect and accept unequal distribution of power (Hofstede, 2001). Democratic institutions typically promote equality, transparency, and participation, which help reduce PD by encouraging more egalitarian relationships between individuals and challenging hierarchical authority (Hofstede, 2001).

In Croatia, the state of democracy has improved over the past 30 years. By 2011, when EU accession negotiations were nearing completion, Croatia had established a relatively stable parliamentary democracy. However, the legacy of high PD remained evident. For example, EU integration negotiations were primarily top-down processes that lacked public support, transparency, and inclusiveness (Skoko, 2016). Since 2013, democratic backsliding has been observed, including political control over the judiciary and limited media independence (Čepo, 2020). The populist surge in 2015 further disrupted political stability. Although political stability was maintained in 2016 and Croatia deepened its EU integration by joining the Schengen Area and Eurozone, these advances came at the cost of suppressing diverse political voices. On the whole, the multi-party and electoral system were implemented, and we hypothesized that*: PD index in Croatia decreased over the pre-accession and post-accession decade (2010–2020)* (*Hypothesis 1a*).

#### 2.5.2 Individualism (IND) variation hypothesis

IND denotes the extent to which society sees people primarily as individuals looking after themselves (high individualism) or primarily as members of communities (high collectivism). Despite a rise in modern values such as individualism and liberalism, collectivism remained prominent in Croatia in the transitional time. The feature was tightly related to the war in the first ten years of transition, which led to the rise of nationalism (Massey et al., 2003).

However, in the process of Europeanization, privatization was accompanied by distinct cultural shifts, most notably the rise of individualism (Pehe and Sommer, 2022). The emergence of private economy was closely associated with ideas of individual agency and taking control of one’s own destiny. Meanwhile, economic development, labor migration, and the shift from extended to nuclear family structures have also contributed to the growth of individualistic values (Soares et al., 2007). Based on the aforementioned points, we hypothesized that *IND index in Croatia increased over the pre-accession and post-accession decade (2010–2020)* (*Hypothesis 1b*).

#### 2.5.3 Masculinity (MAS)/femininity (FEM) variation hypothesis

MAS refers to the degree to which values are associated with stereotypes of masculinity. In high MAS societies people are more assertive and concern less for individual needs and feelings (FEM). According to Hofstede (2001), Croatian society leans toward a nurturing orientation, esteeming factors like quality of life and overall well-being over accomplishments and monetary gain. Wealth is less important because of easier availability, and therefore in Croatia, MAS may have declined between 2010 and 2020, considering the economic development as mentioned above.

The reduction of masculinity could be further emphasized by the booming service industry. In Croatia, the economy growth depends highly on service sector, especially tourism and aggregated services (Šulc and Fuerst-Bjeliš, 2021). A large number of people are net beneficiaries of the tourist growth. The argument would therefore be that tourism provides democratic and egalitarian access to markets, making it less likely to create zero-sum competition. Therefore, it can be presumed that *MAS index in Croatia decreased over the pre-accession and post-accession decade (2010–2020)* (*Hypothesis 1c*).

#### 2.5.4 Uncertainty avoidance (UA) variation hypothesis

UA measures the degree to which members of society are comfortable in unstructured situations. In the first two decades of the transition, Croatian had traumatic experiences with privatization conducted when administrative apparatus unprepared and legal system fragile (Cifrić, 1996). It is therefore understandable if the index of UA was high considering the ambiguities and difficulties.

In the post-accession decade, the UA index may have been lower for the following two reasons: Firstly, the arrival of migrant workers in Croatia may have transformed Croatia into a more diversified society. Secondly, when the economy develops well, the sense of security tends to reduce individuals’ willingness to follow traditional behaviors and conservation values (Inglehart and Baker, 2000; Schwartz, 2014). We assume that Croatians, now belonging to the EU, have become more tolerant of diverse cultures, and so *UA index in Croatia decreased over the pre-accession and post-accession decade (2010–2020)* (*Hypothesis 1d*).

#### 2.5.5 Cultural distance hypothesis

Although national culture may change, the cultural distance between a country and other countries in this process can be stable, diverging, or converging. Croatia is possibly trending toward greater individualism, reduced tolerance for hierarchies, and a stronger emphasis on pluralism. However, these changes are likely to take place simultaneously in other EU members. In this situation, cultures evolve, but they evolve in uniform (Hofstede, 2011), and the cultural distance will stay stable. Cultural distance can even be diverging if cultures change at different speeds and over long periods of time.

However, given the similar social systems and the EU’s cultural integration practices, it is expected that the cultural distance between transition countries and old EU members would narrow. Empirically, Akaliyski (2018) reported that from 2001 to 2008, the newly accepted member states had significantly minimized the cultural gap with the old members. It can be presumed that from 2010 to 2020, the trend will continue. Therefore, Hypothesis 2 is proposed as follows: *The cultural distance between Croatia and other EU members decreased over the pre-accession and post-accession decade (2010–2020).*

The above hypotheses answer the following two questions:

1. How did Croatian cultural values change within 2010–2020? This question is to be addressed by hypotheses 1a–d.
2. Was the cultural value distance shortened between Croatia and the EU members within 2010–2020? This question is to be addressed by hypothesis 2.

## 3 Research method

### 3.1 ESS datasets

To examine the cultural value change over a span of 10 years, we mainly utilized ESS round 5 and 10 datasets collected in 2010/2011 and 2020/2021, respectively. The ESS includes 1,500–2,500 representative samples per country in every round. Beside the data of Croatia, we used the data of 20 EU member states in the ESS datasets as anchors for the study. Following a conventionally used classification method, these members were divided into the old states which joined the EU before 1996, and new states that joined between 2004 and 2013.

### 3.2 Data choosing procedure

To construct latent variables reflecting cultural dimensions, we chose survey questions that while different from those used by Hofstede, still conceptually contain aspects of what Hofstede’s dimensions represent.

PD indicators were chosen with the following consideration: A large PD can be characterized by centralized decision structures, while in the case of small PD the chain of command is not always followed. As in Kaasa et al. (2013), the indicators “Political system allows people to have influence on politics” and “political system allows people to have a say in what government does” were chosen. Moreover, usually in a good democratic society, PD is low, and so the indicators “How satisfied with the way democracy works in country” and “Trust in country’s parliament” were chosen. In the low PD society, people have the chance to participate in social affairs and the process of making decisions are not confined to the minority. Therefore, the indicators “Political system in country ensures everyone fair chance to participate in politics” and “Decisions in country politics are transparent” were chosen.

UA indicators were chosen with the following consideration: Following Kaasa et al. (2013), we chose two indicators showing the importance of behaving properly and the attitude toward sharing customs and traditions: one is “important to do what is told and follow rules” and the other is “important to follow traditions and customs.” The indicators “important to live in secure and safe surroundings” and “important that government is strong and ensures safety” were chosen because they reflect the fact that the population of high uncertainty avoidance societies has a high anxiety level.

The following survey questions were chosen as MAS indicators: “show abilities and be admired,” “get respect,” and “be successful and that people recognize achievements.” These were chosen for being associated with the importance as well as motivation for achievement in masculine societies. In addition, in highly masculine societies, orientation toward money and things prevails over orientation toward people, and earnings are considered to be important. Therefore, the indicator of the importance “to be rich, have money and expensive things” was chosen.

We adopted four variables from Kaasa et al. (2013) as IND indicators: the indicator of the importance to “make one’s own decisions and to be free” reflect aspects of highly individualist societies; a high emphasis on individual initiative is also captured by an indicator of the importance to “think new ideas and being creative.” In addition, individualism is described by the indicators of the importance to “seek fun and things that give pleasure,” and the importance to “have a good time.” These indicators are linked to pleasure and enjoyment that are seen as important in life.

### 3.3 Data analysis

We took four steps to conduct the analysis:

Step one is Factor Analysis. We proceeded with confirmatory factor analysis (CFA) to evaluate the uniqueness of our chosen items in representing their intended dimensions. CFA was conducted using data from ESS 5 and ESS 10 for Croatia and other 20 EU member states. All assumptions were satisfied: Bartlett’s test (*p =* 0), KMO (MSA = 0.86), and the scree plot indicated four factors closely aligned with Hofstede’s cultural dimensions. The reliability of the factors ranged from 0.76 to 0.95, signifying the psychometric reliability of the chosen items (MacCallum et al., 1999). See Table 1 for detailed information of factor structure of the selected ESS items.

**Table 1:** Factor structure of the selected ESS items.

ESS items	Factor 1 (PD)	Factor 2 (MAS)	Factor 3 (UA)	Factor 4 (IND)
Political system allows people to have influence on politics	0.725			
Political system allows people to have a say in what government does	0.741			
How satisfied with the way democracy works in country	0.751			
Trust in country’s parliament	0.757				Political system in country ensures everyone fair chance to participate in politics	0.760				Decisions in country politics are transparent	0.692			
Important to be rich, have money and expensive things		0.549		
Important to get respect from others		0.601		
Important to show abilities and be admired		0.679		
Important to be successful and that people recognize achievements		0.723		
Important to live in secure and safe surroundings			0.564	
Important to follow traditions and customs			0.581	
Important to do what is told and follow rules			0.654	
Important that government is strong and ensures safety			0.553	
Important to seek fun and things that give pleasure				0.765
Important to make one’s own decisions and to be free				0.694	Important to think new ideas and being creative				0.660
Important to have a good time				0.652

PD, power distance; MAS, masculinity; UA, uncertainty avoidance; IND, individualism.

Next, we conducted a correlation analysis. To test the reliability of the ESS culture indicators, we did a correlation analysis of the ESS-based cultural dimensions at the country level with Hofstede’s original findings. Considering that the cultural values of transitional countries might influence the analysis, we utilized only non-transition countries in the ESS datasets for the correlation analysis. It turned out that four dimensions had high correlations, with coefficients ranging as high as 0.47–0.76 for ESS1 and 0.25–0.61 for ESS10. The comparatively lower coefficients concerning ESS 10 can be explained by the changing of culture with the passing of time. See Table 2 for detailed information.

**Table 2:** Correlations between ESS-based factors of cultural dimensions and Hofstede’s national cultural values.

Cultural Dimensions	Dimensions	ESS1	ESS10
Hofstede’s national cultural values	PD	0.63	0.55
	UA	0.58	0.31
	MAS	0.76	0.61
	IND	0.47	0.25

PD, power distance; MAS, masculinity; UA, uncertainty avoidance; IND, individualism.

Step three is to address research question one. We calculated Croatia’s cultural values using the culture indicators from ESS 5 and ESS10 respectively, and conducted a T-test to study variations. We did the same analysis for old EU members to better understand the culture variations of Croatia.

Step four is to address research question two. We studied cultural distance change between Croatia and the other EU members. We initially assessed if the distance of each cultural dimension had decreased or not with Equation 1 below:


(1) $$\;\sqrt[{2}]{{\left(x_{i}-y_{i}\right)}^{2}}\;$$

In Equation 1, *x* and *y* represent two countries, and *i* denotes a specific dimension. The formula calculates the cultural distance between countries *x* and *y* on dimension *i*. Furthermore, we evaluated the multi-dimensional cultural distance between Croatia and the other old EU members, when all dimensions were considered. Since the culture dimensions in Hofstede’s theory can be regarded as a multi-dimensional space, the distance between two countries’ cultures can be measured using the concept of distance between points in multi-dimensional space theory in mathematics, calculated as follows:


(2) $$D=\sqrt{\overset{n}{\underset{i=1}{\sum }}{\left(x_{i}-y_{i}\right)}^{2}}$$

In Equation (2), *D* signifies the distance between two points in a multi-dimensional space; *n* represents the number of dimensions (in the study, *n* = 4); *x* and *y* denote two points in the multi-dimensional space (in the study, different countries); *x_i_* and *y_i_* represent the values of *x* and *y* countries in the dimension *i*. With the above formulas, we calculated the cultural distance between Croatia and the other EU members in 2010 and 2020, respectively. Lastly, we conducted *T*-tests to compare if culture distances between Croatia and the other EU members had decreased within 2010–2020.

## 4 Results

### 4.1 Cultural value change of Croatia within 2010–2020

**Figure 1:**
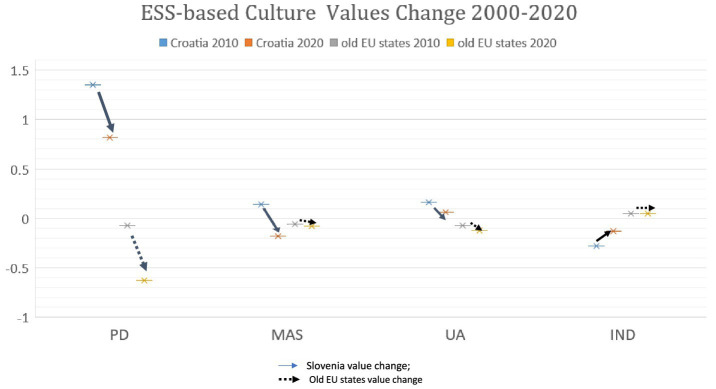
ESS-based cultural value change of Croatia and 12 old EU states (2010–2020).

Figure 1 presents an overview of the variation of cultural values of Croatia during the two survey times. To help interpreting the message, we provided the average cultural value index for the old EU member states (12 observations all together). As shown by Figure 1, in 2010, the mean values of the four culture dimensions for the old EU members were around zero. Compared with the old EU members, in 2010 Croatia had much higher PD (1.35), MAS (0.14), UA (0.16) and lower IND (−0.28).

The comparison between ESS 5 and ESS 10 revealed that ESS-based PD index in Croatia was significantly lowered (by 0.53) in the past decade. So was MAS index, which was lowered by 0.32. IND index was raised by 0.15. UA index had the smallest change, being lowered by 0.10. *T*-tests showed all these culture variations were significant with *p* values near 0.000. The findings agreed with our hypotheses 1a-1d.

We calculated the cultural value changes of the old EU members in the same period and observed that the old EU members also experienced significant (*p* values were from 0.000 to 0.001) decreasing of PD, UA, MAS indexes. The only difference was that the IND index of the old EU states kept stable at 0.05 level in the decade, while IND of Croatia increased significantly. As shown, in 2020, compared with the old EU states Croatia stood out with strikingly high PD index.

### 4.2 Cultural distance change between Croatia and EU member states

To answer question 2, we compared cultural distance between Croatia and 20 EU member states that took part in both ESS 5 and 10. Table 3 presents the results. *T*-tests confirmed that the only significant cultural distance change occurred in IND, which was significantly shortened (*t*-value was 2.831; *p*-value was 0.007). However, for all the other dimensions, no statistically significant differences were observed. Interestingly, an increased PD distance between Croatia and other EU states was implied (*t*-value was 1.792; *p*-value was 0.075), which means that PD was decreasing faster in other EU members than Croatia. The multi-dimensional distance calculated using Equation 2 also demonstrated no significant change (*t*-value was 1.034; *p*-value was 0.308), as depicted in the last row of Table 3. In summary, our findings did not distinctly support Hypothesis 2. Apart from IND, the cultural distance between Croatia and 20 other EU members did not decrease over the 10-year period.

**Table 3:** Summary of cultural value distance variation between Croatia and 20 EU states on ESS-based culture dimensions.

Descriptive Statistics	Distance by indicators of ESS round 5					Distance by indicators of ESS round 10				
	IND	MAS	PD	UA	MD	IND	MAS	PD	UA	MD
Minimum	0.02	0.32	0.21	0.20	0.20	0.27	0.36	0.10	0.20	0.16
Maximum	0.58	0.61	0.92	0.71	1.33	0.50	0.45	0.96	0.46	1.04
Mean	0.30	0.12	0.37	0.15	0.66	0.14	0.03	0.39	0.15	0.56
Standard deviation	0.164	0.279	1.009	0.055	0.070	0.174	0.245	0.870	0.044	0.061
*T*-test if the distance between Croatia and other countries of ESS 10 is equal to the mean of ESS5						∆ (ESS10-ESS5)		*t*-value		*P*-value
Individualism						-0.16		2.831		.007**
Masculinity						-0.09		1.113		.273
Power Distance						0.02		1.792		.075
Uncertainty Avoidance						0.00		0.003		.998
Multi-dimensional distance with Croatia						-0.10		1.034		.308

PD refers to Power Distance; MAS refers to Masculinity; UA refers to Uncertainty Avoidance; IND refers to Individualism. MD refers to multi-dimensional; ** means *p* < 0.01.

As we suspect the unchanged cultural distance might be related to the mixture of the old and new EU states, we compared the values of Croatia with those in the old and the new states, respectively. The result showed that no cultural distance change between Croatia and the new EU states was found. With the old EU states, significantly shortened distance was found, once again, in the IND dimension; the distances with all the other dimensions kept stable. We repeated the calculation with the data of ESS 4 and ESS 9, and the findings were the same: no significantly shortened distance could be found except for the IND dimension.

## 5 Discussion

Our focus was on the cultural value changes in Croatia over a span of 10 years, from pre-accession to post-accession. Two main findings emerged from our study: First, the cultural values in Croatia changed in the same direction as those in the old EU member states. Second, despite this convergence, Croatia maintained the same cultural distance from them.

### 5.1 Changes in Croatian cultural values

All cultural value change hypotheses (1a–1d) were confirmed: PD, MAS, and UA indices decreased, while the IND index increased. Croatian cultural values are shifting in the same direction as those in the older EU member states. This supports Rajh et al. (2016), who found alignment between Croatian and EU cultural values. However, while Rajh et al. (2016) provided a static image, our study traces the trajectory of this transition.

The significant changes in values across all dimensions suggest that the core values promoted by the EU became more prominent in Croatia by 2020. For example, the decline in the UA index points to increased cultural openness and greater tolerance for alternative lifestyles, indicating a more diversified society. Similarly, the decrease in the MAS index suggests that wealth had become less central, reflecting characteristics typical of more developed societies. Given that the alignment between cultural attitudes and formal social institutions is considered a key factor in successful transitions (Nicoara and Boettke, 2015), the past decade has seen a shift in the mentality of Croats toward values hold by citizens of long-standing EU member states.

The rise in Croatia’s IND index and its narrowing gap with other EU states is noteworthy. This supports Bulog et al. (2024), who found that Croatian culture is increasingly oriented toward individualism, reflecting a growing emphasis on personal autonomy and postmaterialist values, typical of technologically and economically advanced societies. This shift is likely driven by steady economic growth and increased socio-economic integration within the EU, particularly through the tourism sector.

However, Croatia’s PD index remained significantly higher than the average of older EU member states. This aligns with Bianchini’s (2014) finding that Croatian elites were reluctant to relinquish their privileges. The authoritarian legacy of the communist era, compounded by the rise of nationalism during the war of independence, could have contributed to weak public demands for democratic and participatory political culture (Finn, 2019; Maldini, 2016). The persistence of high PD can also be attributed to the incomplete political reforms before accession (Szpala, 2011). The EU highlighted issues such as administrative incompetence, widespread corruption, and a judiciary that lacked independence (Szpala, 2011). After EU accession, the absence of internal constraints and external conditionality allowed the ruling party to exploit structural weaknesses in the system.

### 5.2 Cultural value distance between Croatia and other EU members

Our second hypothesis (H2), stating that Croatia could have shortened cultural distance with other EU member states, was not confirmed. Except for IND, the distance of Croatia and the other EU member states had not significantly changed whether in the aspect of individual dimensions (PD, UA, MAS), or multi-dimensional distance.

Our findings can be explained by Hofstede’s statement regarding the stable feature of national culture. Hofstede (2011, p.21) thought for different nations, its cultural position is stable although value is changing constantly. He argued that “…country scores on the dimensions do not provide absolute country positions, but only their positions relative to the other countries in the set.” This statement is still reliable when the transitional country as the case of Croatia is considered. The stable cultural distance between Croatia and other EU members provides empirical evidence that cultural convergence, as envisioned by the EU, is not seen over the 2010–2020 period. The pace of cultural change in Croatia has been insufficient to reduce its distance from other member states.

This result aligns with Akaliyski et al. (2021), who found that while ex-communist nations have increased support for EU values, the change has been slower than in Western Europe. We even observed a not so significantly enlarged distance of PD in Croatia and the other (mainly the old) EU members. Although PD values are lowering in both Croatia and the old EU members, PD in the old is lowering faster. In addition to historical and cultural legacies, this slow rate of change may be explained by waning enthusiasm for EU integration. Once the external pressures of pre-accession faded, internal incentives in Croatia may have been too weak to sustain further reform (Akaliyski et al., 2021; Vasilopoulou and Wagner, 2017).

## 6 Implications and limitations

This study contributes by exploring changes in Croatian cultural values from 2010 to 2020. Over the decade, Croatia experienced significant shifts: decreases in PD, MAS, and UA, alongside an increase in IND. These changes are associated with economic and social development. Individualistic societies tend to exhibit higher innovation and long-term economic growth (Gorodnichenko and Roland, 2017); lower power distance supports the rule of law and governance quality (Kyriacou, 2016); and greater tolerance for uncertainty facilitates legal dispute resolution (Licht et al., 2005).

However, Croatia’s relatively stable cultural distance from other EU members and its high PD index suggest that full “mental integration” into EU values remains incomplete. Despite formal membership, the development of shared values rooted in the free movement of goods and people has progressed slowly (Vasilopoulou and Wagner, 2017; Akaliyski et al., 2021). This highlights the need for greater national and EU-level efforts to promote value convergence. Strengthening common values through coordinated responses to challenges, such as energy crisis or the war in Ukraine, can support this process. Initiatives like student exchanges, joint cultural programs, and policies promoting participation in EU-wide projects can also help bridge cultural gaps.

The second implication concerns cultural research itself. Our findings show that Croatia maintained a stable cultural distance from other EU members between 2010 and 2020. This has important relevance for instructors and practitioners in cross-cultural management and international investment, particularly when assessing cultural similarities and differences. For instance, Croatia’s distinct cultural profile can be used to illustrate EU diversity and to develop teaching materials that help students adapt leadership, negotiation, and communication styles accordingly (Agoraki et al., 2024).

From an economic perspective, cultural differences can significantly impact joint venture performance and the success of mergers and acquisitions. A stable cultural distance enables multinational firms to anticipate potential frictions or synergies, supporting more informed decision-making (Beugelsdijk et al., 2019). Croatia’s consistent cultural profile can therefore help foreign firms develop culturally sensitive strategies and enhance collaboration with Croatian partners.

Caution is needed when interpreting the findings. The primary limitation is the study’s short time span. While Croatia’s relative cultural position remained stable over the past decade, a longer timeframe may reveal cultural convergence. Notably, the IND dimension showed reduced distance during this period already. Other dimensions may also converge if Oshri et al. (2015) are correct in suggesting that support for core EU values increases with each year of membership. Future analyses using upcoming ESS waves can help identify longer-term cultural shifts.

Secondly, given the broad influence of Hofstede’s theory across disciplines, we adopted it as our analytical framework. However, cultural distance indicators derived from the ESS cannot be expected to capture the same phenomena as those based on Hofstede’s original scores. Additionally, events over the past decade, such as the European migration crisis and the COVID-19 pandemic, likely exerted sociocultural effects that may confound observed cultural shifts. Moreover, although the Ukraine conflict, which began in 2022, falls outside our study’s timeframe, it has nonetheless stirred nationalist sentiments in Croatia (Hooghe et al., 2024). Future research is needed to assess whether the ESS-based cultural value measures introduced in the paper remain valid for tracking Croatian value change after 2020.

## Data Availability

The original contributions presented in the study are included in the article/supplementary material, further inquiries can be directed to the corresponding author.
